# Clinical profile of tetanus patients attended at Felege Hiwot Referral Hospital, Northwest Ethiopia: a retrospective cross sectional study

**DOI:** 10.1186/s40064-016-2592-8

**Published:** 2016-06-27

**Authors:** Awoke Derbie, Anteneh Amdu, Amanuel Alamneh, Amare Tadege, Amelwork Solomon, Berhanu Elfu, Daniel Mekonnen, Yinebeb Mezgebu, Seble Worku, Fantahun Biadglegne

**Affiliations:** 1 Department of Medical Microbiology, Immunology and Parasitology, College of Medicine and Health Sciences, Bahir Dar University, P.O. Box 1383 or 79, Bahir Dar, Ethiopia; 2 School of Medicine, College of Medicine and Health Sciences, Bahir Dar University, Bahir Dar, Ethiopia; 3 Department of Epidemiology and Biostatistics, College of Medicine and Health Sciences, Bahir Dar University, Bahir Dar, Ethiopia; 4 Department of Medical Physiology, College of Medicine and Health Sciences, Bahir Dar University, Bahir Dar, Ethiopia; 5 Department of Medical Microbiology, College of Health Sciences, Debre Tabor University, Debre Tabor, Ethiopia

**Keywords:** Tetanus, Case fatality, Immunization, Bahir Dar

## Abstract

**Background:**

Tetanus is an acute, often fatal, disease caused by an exotoxin produced by the bacterium *Clostridium tetani*. It is characterized by generalized rigidity and convulsive spasms of skeletal muscles. Tetanus remains a major public health problem in Ethiopia like other developed nations. The aim of the present study was to assess the clinical profile and outcome of tetanus patients in a referral hospital, Northwest Ethiopia.

**Methods:**

This is a retrospective cross sectional study in which we collected, compiled and analyzed medical records of patients aged greater than 15 years who were admitted at Felege Hiwot Referral Hospital from Sep 2012 to Sep 2015. Tetanus was diagnosed using clinical observations. Data were entered, cleared, and analyzed using SPSS statistical software package.

**Results:**

Among 110 tetanus cases 84 (76.4 %) were males. Trauma was the most common antecedent cause. Most of the patients had no history of tetanus toxoid immunization. Aspiration pneumonia at 34.5 % and dysautonomia at 11.8 % were found to be the most frequently observed complications. About 36 (32.7 %) patients were died due to tetanus and the most common immediate cause of death was respiratory failure at (83.3 %). Patients’ area of residence (p = 0.004), type of complications (p = 0.00) and severity of tetanus (p = 0.00) were found statistically associated with the type of treatment outcomes.

**Conclusions:**

In the study, the case-fatality rate was found to be very high. Therefore, there is a need to universal tetanus immunization and health information dissemination.

## Background

Tetanus, an infective intoxication of the nervous system by *Clostridium tetani*, is an ancient disease which is associated with a high mortality rate. Despite the widespread availability of a safe and effective vaccine against this disease, it remains a major health problem in developing countries, and also occurs in countries with good standard of medical practice (Anuradha 2006; Ogunrin 2009).

Transmission is primarily by contaminated wounds (apparent and inapparent). *C. tetani* spores are found in soil and in animal and human faeces (Abrutyn 2008). Tetanus is a neurological disorder, characterized by increased muscle tone and spasms. Characteristically, early spasms of the facial muscles (“lockjaw”) are followed by spasm of the back muscles and sudden, generalized tonic seizures (Abrutyn^2008^; Ogunrin 2009).

The majority of the tetanus cases occur in the developing countries where it is associated with high mortality ranging from 40 to 60 % due to the lack of effective immunization, poor treatment of injuries and decline in protective antibodies. The mortality rate is much lower in developed countries due to the availability of facilities for intensive care, unlike in the developing countries (Anuradha 2006; Chukwubike and God Spower 2009; Khaskheli et al. 2013; Tadesse and Gebre-Selassi 2009). The World Health Organization (WHO) global and regional immunization profile report, the number of reported all forms of tetanus cases by the year 2014 in European region was found to be 67 but it was 2900 in Africa (WHO 2015a, b). This is likely to be a huge understatement.

Tetanus is classically divided into four clinical types: generalized, localized, cephalic, and neonatal. These are valuable diagnostic and prognostic distinctions, but they reflect host factors and the site of inoculation rather than differences in toxin action. Clinical findings showed that there are three different forms of tetanus namely; local, cephalic and generalized tetanus (WHO 2015a, b, c). It typically presents with trismus, difficulty in swallowing and more ominously difficulty in breathing or opisthotonus (Ogunrin 2009).

The diagnosis is entirely clinical and does not depend upon bacteriologic confirmation. The management of tetanus patients is too demanding, prolonged, and expensive both in terms of materials and manpower. The goal of therapy to tetanus cases is to eliminate the source of toxin, neutralize unbound toxin, and prevent muscle spasms while monitoring the patient’s condition and providing support, especially respiratory support until recovery (Ogunrin 2009; CDC 2015; WHO 2015a, c).

In Ethiopia, the prevalence and incidence of tetanus and its associated complications, control of the disease is far from adequate. Therefore, the aim of this study was to assess the demographic and clinical profile of tetanus patients at FHRH above the pediatric age group.

## Methods

### Study design, area, period and subjects

Medical records of tetanus patients were analyzed retrospectively to assess the demographic and clinical profile of 110 adult tetanus patients registered from 30 Sep 2012 to 30 Sep 2015 in the department of internal medicine at FHRH, which is one of the referral hospitals in Amhara Regional State, Bahir Dar-Ethiopia. All complete data registered during the study period were taken from hospital records for analysis. Tetanus was diagnosed by clinical observations.

### Data collection and analysis

The data was collected by retrospective examination of patient records using data extraction sheet. Patient’s demographic and clinical data such as; age, sex residence, TT immunization status, antecedent cause of tetanus, severity, complications, and outcome of tetanus were extracted for analysis. All data were entered, cleared, and analyzed using SPSS version 20 (IBM Corp. Released 2011. IBM SPSS Statistics for Windows, Version 20.0. Armonk, NY: IBM Corp.). Descriptive statistics was used to present data and Chi square test was employed to assess association between tetanus treatment outcomes with relevant variables. Level of statistical significance was set at p value less than 0.05.

### Ethical issue

Institutional ethical permission was obtained from the Research and Publication Committee Ethical Review Board of Bahir Dar University.

## Results

### Demographic data

Of the total registered tetanus patients 84 (76.4 %) were males. The mean age of the patients was 43.4 ± 15.1 years. Of the registered study subjects, 66 (60 %) of them were from rural areas. Among the study subjects, 17 (15.5 %) of the patients had prior TT immunization history, while the majority of them, 93 (84.5 %) did not know their immunization status. Trauma at 66 (60 %) of the cases was the most common causes of tetanus. Forty (36.4 %) patients arrived to the hospital with in 72 h after the antecedent cause. Out of the 84 tetanus patients 9 (10.7 %) took TAT prophylaxis (Table 1). Of these nine patients, 5 (55.6 %) of them had severe form of tetanus and 2 (22.2 %) of them died in the course of their management as compared with those patients who didn’t take TAT who end up with severe form of tetanus, 21 (53.8 %) and death, 15 (38.5 %).TAT prophylaxis didn’t show statistical significance with severity and outcome of tetanus (p > 0.05).

**Table 1:** Demographic and related clinical data of tetanus patients beyond pediatric age group attended at FHRH, Bahir Dar, 2015

Variables	Frequency (%)	
Sex		
Male	84 (76.4)	M:F ration 3.2:1
Female	26 (23.6)	
Age category		
15–25	15 (13.6)	Mean: 43.4 years SD: ±15.1
26–35	22 (20.0)	
36–45	31 (28.2)	
46–55	14 (12.7)	
56–65	20 (18.2)	
>65	8 (7.3)	
Residence		
Rural	66 (60)	
Urban	44 (40)	
TT vaccination history		
Yes	17 (15.5)	
No	16 (14.5)	
Unknown	77 (70.0)	
Antecedent causes		
Trauma	66 (60.0)	
Burn	3 (2.7)	
Surgery	3 (2.7)	
Devitalized tissue	5 (4.5)	
Co infection	4 (3.6)	
Cryptogenic	24 (21.8)	
Traditional practice	5 (4.5)	
TAT prophylaxis given		
Yes	9 (10.7)	
No	39 (46.4)	
Unknown	36 (42.9)	

### Clinical profile of tetanus patients

Among the study subjects, 103 (93.6 %) were diagnosed with generalized tetanus during the time of admission. According to *Ablett* classification of severity of tetanus (Ogunrin 2009), overall 24 (21.8 %), 31 (28.2 %) and 55 (50 %) patients were presented with mild, moderate and severe form of tetanus respectively. All patients diagnosed with tetanus have got the standard management protocol (TAT, muscle relaxant, antibiotics, put on dark room). Furthermore, 13 (11.8 %) patients’ received debridement procedure and a patient was transferred to intensive care unit (ICU) and was mechanically ventilated by intubation (oropharyngeal). Some of the study subjects encountered complications following tetanus. Aspiration pneumonia, at 36 (34.5 %) and autonomic dysfunction (dysautonomia) at 13 (11.8 %) were the most frequently observed complications followed by upper airway obstruction at 6 (5.5 %).

Of all managed patients, 36 (32.7 %) died, 37 (33.6 %) survival to hospital discharge, 37 (33.6 %) patients discharged themselves against medical advice for unspecified reason. Most of the patients (80.6 %) died within 1 week during their stay in the hospital indicating that patients did not get adequate respiratory support. The two most common cause of death were respiratory failure at (83.3 %) and multi organ failure at (16.7 %) (Table 2). Management outcome of tetanus patients showed significant statistical association with patients area of residence (p = 0.004), type of complications (p = 0.000) and severity of tetanus (p = 0.000) (Table 3).

**Table 2:** Clinical profile of tetanus patients attended at FHRH, Bahir Dar, 2015

Variables	Frequency (%)
Clinical presentation	
Local	3 (2.7)
Cephalic	4 (3.6)
Generalized	103 (93.6)
Onset of symptoms after injury	
<3 days	40 (36.4)
3–14 days	35 (31.8)
>14 days	13 (11.8)
Unknown	22 (20.0)
Managements given	
Standard Mgt	110 (100)
Debridement	13 (11.8)
Oropharyngeal intubation	1 (0.9)
Complications	
Aspiration pneumonia	38 (34.5)
Autonomic dysfunction	13 (11.8)
Air way obstruction	6 (5.5)
Renal failure	2 (1.8)
Decubitus ulcer	2 (1.8)
None	49 (44.6)
Severity	
Mild	24 (21.8)
Moderate	31 (28.2)
Severe	55 (50.0)
Management outcome	
Death	36 (32.7)
Survival to hospital discharge	37 (33.6)
Against medical advice	37 (33.6)
Causes of death	
Respiratory failure	30 (83.33)
MOF	6 (16.67)

*MOF* multi organ failure

**Table 3:** Tetanus management outcome of study subjects at FHRH, Bahir Dar, 2015

Variables	Management outcome				*p* value
	Death	Survival to hospital discharge	Against medical advice	Total	
Sex					
Male	29	27	28	84	0.742
Female	7	10	9	26	
Residence					
Urban	14	22	8	44	*0.004*
Rural	22	15	29	66	
TT vaccination history					
Yes	4	8	5	17	0.428
No	4	4	8	16	
Unknown	28	25	24	77	
TAT prophylaxis history					
Yes	2	4	3	9	0.856
No	15	12	12	39	
Unknown	11	14	11	36	
Not required	8	7	11	26	
Antecedent cause of tetanus					
Trauma	20	25	21	66	*0.094*
Burn	2	1	0	3	
Surgery	0	1	2	3	
Devitalized tissue	3	1	1	5	
Co infection	0	4	0	4	
Cryptogenic	10	4	10	24	
Traditional practice	1	1	3	5	
Clinical presentation					
Local	2	1	0	3	0.217
Cephalic	1	3	0	4	
Generalized	33	33	37	103	
Time gap between accident and presentation to the hospital					
<3 days	9	19	12	40	0.203
3–14 days	12	12	11	35	
>14 days	5	3	5	13	
Unknown	10	3	9	22	
Complications					
Aspiration pneumonia	22	5	11	38	*0.000*
Autonomic dysfunction	2	6	5	13	
Air way obstruction	6	0	0	6	
Renal failure	2	0	0	2	
Decubitus ulcer	0	1	1	2	
No complications	4	25	30	59	
Severity of tetanus					
Mild	1	23	0	24	*0.000*
Moderate	5	12	14	31	
Severe	30	2	23	55	

## Discussion

Regardless of efforts made by the WHO, tetanus remains a global health problem. Lack of immunization, low level of education, poor wound management and personal hygiene increased the burden of tetanus in developing countries (Oladiran et al. 2002). In addition, high incidence of tetanus admissions in developing countries has been attributed to low levels of health awareness in terms of vaccination and availability of human and material resources to manage the disease (Amare et al. 2012). Researchers showed that mortality due to tetanus could be reduced intensified immunization efforts by all health care providers in the developing nations (Oladiran et al. 2002; Woldeamanuel 2012).

The burden of tetanus is high only in resource limited countries like Ethiopia. Agrarian rural life with limited vaccine typifies tetanus risk in Ethiopia (Woldeamanuel 2012). At present, the country is involved in expanding tetanus control trends through infant immunization and eliminating highly prevalent maternal and neonatal tetanus (MNT) (Woldeamanuel 2012).

High incidence of tetanus admissions in developing countries like Ethiopia is attributed to low levels of health awareness in terms of vaccination and availability of human and material resources to manage the disease (Amare et al. 2012; Woldeamanuel 2012).

In this study majority of the tetanus patients were males and rural dwellers. Similarly, the most common portal of entry among patients in this study was trauma, which constituted 60 % of all the admitted tetanus patients. Similar finding was reported by a study from India (Anuradha 2006).

In the present study, 84.5 % of the tetanus patients had no prior tetanus immunization. This might be due to low coverage of tetanus vaccination in the study area. Furthermore the finding showed that 10.7 % of the patients took TAT prophylaxis. The immunization status of adults in the third world countries like Ethiopia is quite limited. The primary immunization, including maternal immunization coverage and addressing the recommended booster dose for candidates is inadequate (Amare et al. 2012).

In the present study, 21.8, 28.2 and 50 % of the tetanus patients had mild, moderate and severe form of tetanus during the time of presentation respectively. Comparable finding was reported by other studies (An et al. 2015; Tadesse and Gebre-Selassi 2009). Aspiration pneumonia at 34.5 % and dysautonomia at 11.8 % were the most frequently observed complications in our study. These results are consistent with others (Amare et al. 2012; Anuradha 2006). Researchers showed that the use of intensive treatment for tetanus patients decreased the incidence of respiratory complications (Chukwubike and God Spower 2009; Oshinaike et al. 2012; Tadesse and Gebre-Selassi 2009). Prior to the introduction of intensive care unit (ICU) including ventilation, 80 % of deaths due to tetanus were related to acute respiratory failure (Oshinaike et al. 2012; Tadesse and Gebre-Selassi 2009).

In the present study, respiratory failure at 83.3 % was found to be one of the principal immediate causes of death, which is in agreement with other studies (Amare and Yami 2011; An et al. 2015; Tadesse and Gebre-Selassi 2009). This might be due to availability of very limited ICU services in the country. In this study the reported mortality rate was at 32.7 %. Likewise, studies conducted elsewhere in Ethiopia reported the same trend ranging from 27 to 41.7 % (Amare et al. 2012; Amare and Yami 2011; Hodes and Teferedegne 1990; Tadesse and Gebre-Selassi 2009). Furthermore, comparable findings were reported in Nigeria ranging from 42.9 to 56.2 % (Chukwubike and God Spower 2009; Oladiran et al. 2002) and India at 37.8 % (Anuradha 2006). In contrast relatively low fatality rate at 6.4 % was reported in Vietnam (An et al. 2015), may be due to large scale availability of safe and effective vaccine in this country.

About 37 (33.6 %) patients discharged themselves against medical advice for unspecified reason. These patients might be less likely to survive and this obviously would imply that the outcome of tetanus could be worse than reported in this paper.

The main limitations of our study were, the study lacks detailed clinical data and analysis to identify predictors of mortality. This calls for the improvement in documentation and keeping of medical records of patients properly. However, our study is one of the few that provides baseline information concerning Tetanus patients at FHRH, northwest Ethiopia, and this could be useful for planning appropriate Tetanus control strategies, especially in the present study areas and in settings where Tetanus is common.

## Conclusions and recommendations

In conclusion, high case fatality rate at 33 % was documented due to tetanus in the studied area. Efforts to identify complications early in the course of the disease will help to recognize patients that will need more intensive treatment. Widespread use of appropriate vaccination and prophylaxis is essential to prevent tetanus. Consequently, continuous and intensified public health strategies on health education as well as universal and adequate coverage of immunization program are required.

## Abbreviations

TT: Tetanus toxoid
TAT: Tetanus Anti Toxin
ICU: Intensive Care Unit
MNT: Maternal and Neonatal Tetanus
WHO: World Health Organization
CDC: Center for Disease Control
FHRH: Felege Hiwot Referral Hospital
