# Erratum to: Clinical profile of tetanus patients attended at Felege Hiwot Referral Hospital, Northwest Ethiopia: a retrospective cross sectional study

**DOI:** 10.1186/s40064-016-2803-3

**Published:** 2016-07-27

**Authors:** Awoke Derbie, Anteneh Amdu, Amanuel Alamneh, Amare Tadege, Amelwork Solomon, Berhanu Elfu, Daniel Mekonnen, Yinebeb Mezgebu, Seble Worku, Fantahun Biadglegne

**Affiliations:** 1 Department of Medical Microbiology, Immunology and Parasitology, College of Medicine and Health Sciences, Bahir Dar University, P.O. Box 1383 or 79, Bahir Dar, Ethiopia; 2 School of Medicine, College of Medicine and Health Sciences, Bahir Dar University, Bahir Dar, Ethiopia; 3 Department of Epidemiology and Biostatistics, College of Medicine and Health Sciences, Bahir Dar University, Bahir Dar, Ethiopia; 4 Department of Medical Physiology, College of Medicine and Health Sciences, Bahir Dar University, Bahir Dar, Ethiopia; 5 Department of Medical Microbiology, College of Health Sciences, Debre Tabor University, Debre Tabor, Ethiopia

## Erratum to: SpringerPlus (2016) 5:892 DOI 10.1186/s40064-016-2592-8

Upon publication of the original article (Derbie et al. 2016), it was noticed that Berhanu Elfu’s name was incorrectly written as ‘Bihanu Elfu’. This has now been corrected in this erratum and updated in the original article.

## References

[CR1] Derbie A, Amdu A, Alamneh A, Tadege A, Solomon A, Elfu B, Mekonnen D, Mezgebu Y, Worku S, Biadglegne F (2016). Clinical profile of tetanus patients attended at Felege Hiwot Referral Hospital, Northwest Ethiopia: a retrospective cross sectional study. SpringerPlus.

